# Coding Public Health Interventions for Health Technology Assessments: A Pilot Experience With WHO's International Classification of Health Interventions (ICHI)

**DOI:** 10.3389/fpubh.2021.620637

**Published:** 2021-06-16

**Authors:** Markus Wübbeler, Sebastian Geis, Jovana Stojanovic, Lise Elliott, Iñaki Gutierrez-Ibarluzea, Irene Lenoir-Wijnkoop

**Affiliations:** ^1^Department of Nursing Science, University of Applied Sciences, Bochum, Germany; ^2^Department of Health, Kinesiology, and Applied Physiology, Faculty of Arts and Science, Concordia University, Montreal, QC, Canada; ^3^Montreal Behavioural Medicine Centre, CIUSSS du Nord-de-l'Ile-de-Montréal, Montréal, QC, Canada; ^4^Centre for Guidelines, National Institute for Health and Care Excellence, Manchester, United Kingdom; ^5^Basque Foundation for Health Innovation and Research (BIOEF), Barakaldo, Spain; ^6^Basque Office for HTA (Osteba), Barakaldo, Spain; ^7^Utrecht Institute for Pharmaceutical Sciences, Utrecht University, Utrecht, Netherlands

**Keywords:** public health intervention, International Classification of Health Interventions, Health Technology Assessment, coding, feasibility evaluation

## Abstract

**Introduction:** An important requirement for successful public health interventions is a standardized classification in order to make these health technologies comparable in all contexts and recognized by all parties. The WHO International Classification of Health Interventions (ICHI), including an integrated public health component, has been developed to propose such an international standard.

**Methods:** To test (a) the translation of public health interventions to ICHI codes and (b) the technical handling and general coding in public health, we used a set of public health interventions from a recent cross-sectional survey among Health Technology Assessment professionals.

**Results:** Our study showed that handling of the ICHI interface is stable, that there is a need for specificity and adequate detail of intervention descriptions and desired outcomes to code adequately with ICHI and that the professional background of the coder, as well as his/her sex might influence the selection of codes.

**Conclusion:** International Classification of Health Interventions provides a good coverage of public health interventions. However, the broader character of system wide interventions, often involving a variety of institutions and stakeholders, may present a challenge to the application of ICHI coding. Based on this experience, we would tailor future surveys more specifically to the needs of the classification and we advise training for health professionals before coding with ICHI. Standards of reporting will likely strengthen insights about the efficiency of primary prevention interventions and thus benefit long-term health of populations and structured HTA reporting process.

## Introduction

There is a growing interest in assessing Public Health Interventions (PHIs) from a Health Technology Assessment (HTA) perspective. A joint action of the Society Health Technology Assessment international (HTAi) and the International Network of Agencies for Health Technology Assessment, which was endorsed by many organizations alike, has recently elaborated and published a new definition of HTA: “*HTA is a multidisciplinary process that uses explicit methods to determine the value of a health technology at different points in its lifecycle*.” The purpose is to inform decision-making in order to promote an equitable, efficient, and high-quality health system (1).

A health technology is an intervention developed to prevent, diagnose or treat medical conditions; promote health; provide rehabilitation; or organize healthcare delivery. The intervention can be a test, device, medicine, vaccine, and may also include a procedure, program, or system related to the management of public health concerns. In accordance to the widespread and broadly accepted definition (2), PHIs are considered a health technology. Different authors have pointed out that HTA organizations do not frequently include PHIs among their portfolio of technologies to be assessed (3, 4). Nevertheless, HTA agencies and units are increasingly including PHIs among the main topics of discussion and projects such as the HTAi's Interest Group on Public Health (5) and the European Public Health Association's HTA devoted group (6) with over 1,000 members. Moreover, the COVID-19 pandemic highlights the importance of public health interventions to all countries worldwide, increasing demand for evidence-based interventions to control a further outbreak of the coronavirus disease or future pandemics.

However, due to their complexity (7), there are some challenges to be overcome when assessing PHIs from the HTA point of view. One of those is that studies evaluating PHIs are of lower methodological rigor, being in most cases non-randomized studies, often published as gray literature. Furthermore, assessment frameworks including their allowance of experts opinions do not fit with common processes and methods for HTA (7).

Even so, it is important to note that HTA methods are formal, systematic, and transparent, using state-of-the-art methodology to select the best available evidence (1). It begins with the well-known PICO question in which Patients or Population, Intervention, Comparison and Outcomes need to be defined (8). A question is the starting point for any HTA report of quality. This means that the question will feature the characteristics of Patients or Population as accurately as possible, the Intervention will be described in full-detail, in order to find the suitable Comparator or standard of practice and the Outcomes will be chosen including the perspective of the different stakeholders and will be justified according to the context in which the intervention is applied. Bearing in mind all the aforementioned aspects, there is a clear necessity that anyone who refers to an intervention is talking about the same concept. Supporting such a harmonized understanding would also make the search for and pursuit of the evidence base more accessible to clinicians and researchers. This requires a standardized classification of health interventions in order to make them comparable in all contexts and recognized by all parties.

Classifications in health care have always been a matter of controversy and they are under continuous discussion and development. There is a general acceptance of the International Classification of Diseases (ICD) which is maintained by the World Health Organization (WHO) and currently in its 10th version. Its 11th revision, the ICD-11, was accepted by WHO's World Health Assembly (WHA) in May 2019 and will officially come into effect in January 2022. Another widespread classification method is the International Classification of Functioning, Disability and Health (ICF) which is a method to classify health and health-related domains (9). While this has provided a way to harmonize the description of diseases and health dimensions, these classifications did not solve the issue that HTA poses, namely the agreed and accepted definition of interventions. This changed in 2017 when a new way of classifying interventions was proposed, the International Classification of Health interventions (ICHI) (10). The ICHI is currently being developed to provide a common tool for reporting and analyzing health interventions (11). After finalization, it is intended to join the ICD and ICF as a WHO-FIC reference classification. It embraces interventions on: diagnostics, medical therapies, surgical procedures, mental health, primary care, allied health, functioning support, rehabilitation, traditional medicine and -last but not least- public health. In order to address the specific needs worldwide, ICHI was designed with low complexity to be applicable in countries without procedure classification (12) and should provide a foundation for optimizing data management and the ability to compare health interventions in various contexts and settings worldwide. Furthermore, in the case of PHIs, ICHI categorization may be useful for stakeholders engaged in HTA reports by disentangling the complexity of these interventions as well as establishing a common HTA framework for their analysis. Based on this classification that will be translated in almost all languages and probably adopted by most systems, we have analyzed HTA reports on PHIs retrieved from a previous survey in which 76 PHIs were identified from 2013 to 2018 (5).

We aimed to test the translation of PHI descriptions, coming from a cross-sectional survey of HTA professionals toward a standardized language (ICHI) for healthcare interventions. Moreover, we aim to evaluate the ICHI classification handling for health professionals, with an emphasis on technical handling and coding procedures.

## Materials and Methods

### Structure of the ICHI

International Classification of Health Interventions identifies more than 7,000 interventions (13), covering 27 chapters and four sections (Body Systems and Functions, Activities and Participation Domains, Environment, and Health-related Behaviors) (10). Each intervention is represented by a title including an individual seven-character code, called “stem code” that covers three axes: three characters to describe the so-called Target (i.e., entity on which the Action is carried out; unit on which the intervention is carried out), two characters for the Action (i.e., deed done by an actor to a Target) and two characters for the Means (i.e., processes and methods by which the Action is carried out). The axes are defined as follows (14).

Example: VAB.WF.QE - Enforcement of legislation or regulations for restrictions or requirements concerning the consumption or use of tobacco products

Target: VAB Tobacco use behaviors

Action: WF Restrictions on the consumption/use of products

Means: QE Enforcement

Each stem code has a unique combination of categories from these three axes. The current ICHI version provides a number of information fields to verify the accuracy of the stem code as shown in Table 1.

**Table 1:** ICHI coding information.

**Category**	**Information value**
Definition	Provides a description of the intervention
Index terms	Gives examples of terms that should be classified to that specific stem code
Includes notes	Is used to define the scope of a stem code
Code also	Used to advise the user that an additional code may be assigned when a certain intervention is selected
Excludes notes	Lists specific interventions that are classified elsewhere in ICHI

#### Data Collection

A cross-sectional survey, distributed among international societies and institutions involved in HTA, collected data on what kind of PHIs have been assessed in the period from 2013 to 2018 as well as details of the technology/intervention. The survey was sent out to a total of 85 recipients. In total, 52 respondents from all continents answered the survey. The majority came from European countries (34%), followed by North American (26.9%) and South American countries (19.2%) (5). The obtained information was entered in a standard data collection extraction spread sheet (Microsoft Excel, version 2016) and categorized according to the ICHI.

#### Data Coding

The coding and validation procedures were carried out in the timeframe from November 2019 to March 2020. Initially, the coding was performed by a pair of two independent researchers from the University of Applied Sciences in Bochum (MW, SG). Further to that a second round of coding was undertaken by a collaborator from the National Institute for Health and Care Excellence in the UK, experienced in the Public Sector with solid experience in Data Analysis (LE). Next, to ensure consistency between the two sets of codes, the compiled spread sheet was analyzed by another pair of independent researchers (JS, ILW). This third round consisted of cross-check including comments and discussion of discrepancies between the sets until final consensual agreement on coding outcomes was reached. The ICHI coding book was accessed using the online version of the ICHI database, currently available in a beta-3 stage and presented as an HTML website hosted by the German Institute of Medical Documentation and Information (https://mitel.dimi.uniud.it/ichi/). For the time being, ICHI is provided in English language only. In the absence of an automated procedure through a data export option for ICHI, categorization was done manually by searching for ICHI interventions and procedures using the search box presented on the website. Terms used for the search were predefined by the free text answers given by the survey participants. Where a search did not lead to an appropriate coding option, either due to no results presented or unsuitable codes, the coding tree option, provided on the website was used. This equals a selecting process using the main axes of ICHI (target, action, means), hand selecting main- and subcategories until a suitable fit. Where needed, in case of uncertainty on an intervention or -in some cases- to ascertain the nature of a clinical procedures or to check the purpose of a drug, the point was resolved either through research on the internet or discussion with a clinical colleague.

Due to the lack of a data export option for ICHI, the Excel spreadsheet was completed with the matching ICHI codes that were selected, including the descriptor and definition for every code potentially relevant to the recorded interventions. To reduce bias in hand coding, the ICHI codes were cross-checked with survey answers. This step was particularly relevant to maintain data quality in cases where multiple ICHI codes could be assigned to a free text answer.

For validating of the final categorization, a new spreadsheet was created, where each code was represented in one line (Supplementary Material). This allowed the identification of similarities and discrepancies at a glance.

## Results

Our analysis yielded a total of 75 PH technologies, as one intervention was ultimately excluded from the ICHI categorization process, due to low reporting of the intervention specifics (Supplementary Material). The outcomes could be subdivided into the following groups: primary prevention (42.1%), secondary prevention (48.7%), tertiary prevention (5.3%), and other (3.9%). Using a plain categorization of the most frequent health concerns showed screening of chronic diseases (25%) as the most frequent intervention, followed by infectious diseases prevention (21.1%) and maternal, pre- and neonatal screening initiatives (9.2%). The lowest rates were found for environmental interventions such as tobacco cessation (3.9%) and mental health screening (3.9%). The most frequent codes categorized to the survey answers are shown in Table 2.

**Table 2:** Answers coded with WHO international classification of health (ICHI).

**Coding**	**Descriptors**	***N***
VDB.VC.ZZ	Public health surveillance concerning screening behaviors	18
DTB.DB.AE	Percutaneous administration of immunological agent, not elsewhere classified	12
UA1.VC.ZZ	Public health surveillance concerning products and technology	9
UBC.VC.ZZ	Population public health surveillance	9
VDA.VC.ZZ	Public health surveillance concerning immunization behaviors	8
VEF.VC.ZZ	Public health surveillance concerning sexual behaviors	7
NMF.AH.AC	Cervical papanicolaou smear	6
UA1.WG.QF	Economic incentives concerning products and technology in relation to health	6
VDB.WG.QF	Economic incentives to encourage improved health behaviors relating to use of health screening services	6
PYA.PP.ZZ	Genetic counseling	5
LCA.BA.BA	Mammography	4
UAC.AA.ZZ	Assessment of medication	4
UEP.VE.ZZ	Health care infection control measures	4
VAB.RD.ZZ	Provision of products to support improved health behaviors relating to tobacco use, not elsewhere classified	4
VEF.AA.ZZ	Assessment of sexual behaviors	4

### Technical Handling

The handling of the HTML interface of ICHI was stable, no software bugs or unexpected reloads of the website were noticed. Navigation on the website, in terms of the interface design, did not lead to a poor user experience and therefore worked well. The website provides an introduction manual to describe the use of ICHI including coding rules. Examples of ICHI coding options are shown in Table 3.

**Table 3:** Examples of ICHI coding options.

**Nr**	Public health intervention^*^	Intended ouctome^*^	**ICHI coding options**	**ICHI descriptor**
1	Down syndrome screening	Screening	NMR.AA.BJ	Ultrasound assessment for detection of fetal abnormality
			NMR.AA.ZZ	Assessment of fetal or embryonic structure
2	Pre-/postnatal mother-child care	Reaching deprived population groups	SSK.PM.ZZ	Education about parent-child relationships
			SSK.RB.ZZ	Practical support for parent-child relationships
			VEJ.PH.ZZ	Training to influence parenting behaviors
			VEJ.PM.ZZ	Education to influence parenting behaviors
			VEJ.PN.ZZ	Advising about parenting behaviors
			VEJ.PP.ZZ	Counseling about parenting behaviors
			VEJ.RC.ZZ	Emotional support for parenting behaviors
			VEJ.VB.ZZ	Awareness raising to influence parenting behaviors
3	HPV screening	Clinical and cost-effectiveness	NMF.AH.AC	Cervical papanicolaou smear
			UBC.VC.ZZ	Population public health surveillance
			VDB.WG.QF	Economic incentives to encourage improved health behaviors relating to use of health screening services
			VDB.VC.ZZ	Public health surveillance concerning screening behaviors
			VEF.VC.ZZ	Public health surveillance concerning sexual behaviors
4	Parent-Child Assistance Program for Preventing Fetal Alcohol Spectrum Disorder	FASD cases prevented	VAA.PH.ZZ	Training to influence alcohol use behaviors
			VAA.PM.ZZ	Education to influence alcohol use behaviors
			VAA.PN.ZZ	Advising about alcohol use behaviors
			VAA.PP.ZZ	Counseling about alcohol use behaviors

* *cross-sectional survey answers*.

### Coder Agreement—Range of Codings

The 75 PH interventions resulted into 280 ICHI coding options, ranged from 1 up to 12 ICHI codes potentially relevant to code a single intervention (Supplementary Material). PH technologies with a wide range of coding options were “interventions preventing cannabis use among high school” with 12 options and “programs to prevent obesity in adolescents” with 10 options. While the target might be clear, like VAC (illicit drug use behaviors) at the beginning of a code, the range of potential actions lead to a variance of suitable codes. To highlight the knowledge and qualifications of a person, the ICHI classification distinguishes between advising, education, training and counseling (see example of coding options):

- PN—Advising: Recommending a course of action in relation to changing or maintaining functioning, environment or behavior.
- PM—Education: Providing information to improve knowledge. Education may be of the parent or carer of a person or the person themselves.
- PH—Training: Teaching, enhancing or developing skills through practice
- PP—Counseling: Providing therapeutic or supportive communication

The absence of detail with some of the interventions of the initial survey meant that they were often difficult to code, which was sometimes exacerbated by a concomitant lack of detail on the intervention outcome that survey participants were asked to provide. A typical example of this was the intervention “*pre-/post-natal mother-child care*” (under #2 in the Supplementary Material), which had a “reaching deprived population groups” outcome and as this outcome was somewhat generic, the codes relating to “advising,” “counseling,” “education” and “training” were all potentially relevant; but made it impossible to differentiate between them. Another example, under #8, “*pre-exposure prophylaxis (PrEP)*,” did not have a recorded outcome and as such presented a similar problem in relation to differentiation. A further example highlighting the same issue; i.e., the impossibility of differentiating codes relating to “advising,” “counseling,” “education,” and “training” was “*Interventions preventing cannabis*” *use among high school* [see Supplementary Material (#31)]. The stated outcome was “Cannabis use,” which perhaps could have been more accurately described as “prevention of cannabis use.” The above highlights the need for precise and adequate details of intervention descriptions and desired outcomes to code adequately with ICHI.

### Coder Agreement—Disentangling Terminology

The potential difference in the use of terminology also seemed to be a factor in the code selection of coders, as illustrated by #32, “*Programs to prevent obesity in adolescents*,” which had a stated outcome of “Effectiveness.” In this example, some coders selected codes relating to eating behaviors, whilst another coder also selected codes relating to support for weight management.

Another example, described as “*Smoking cessation interventions*” (#68), had a stated outcome of “Cost-utility; smoking-related morbidity and mortality.” In this example, like the other, coders were united in some of their code choices; specifically, around codes relating to tobacco use behaviors. However, there appeared to be differences between coders in their interpretation of the cost-utility element of the outcome; which consequently influenced code selection.

These findings highlight the importance of clarity on the details of the reported intervention and the outcome. Moreover, differences in understanding or interpretation, by coders, are very likely to influence code selection and increase the incidence of code divergence.

### Coder Agreement—Influence of the Coders Background and Sex

The background of the coder seemed, on occasions to have an influence on the codes selected for the intervention. A typical example of this was #30, “*Lung cancer screening*,” which had an outcome of “appropriateness of implementation.” An identical code was selected by two coders; but of the remaining codes, one set of coders selected codes that related to public health surveillance, whereas the other coder selected codes with a more clinical focus. We experienced differences between researchers with a clinical background and those with a more public health focus.

The sex of a coder may also influence their choice of code for some interventions. A typical example of this was *HPV-vaccination* (#25), which had an outcome of “decrease of cervix carcinoma.” All coders selected the code that related to the actual vaccination; that is, “*Percutaneous administration of immunological agent*.” However, the female coders selected codes relating to public health surveillance of products/technology and sexual behavior. Whereas, the male coder chose codes relating to counseling on health-related behaviors and the assessment of the urogenital system.

## Discussion

We aimed to evaluate the transferability of PH intervention data from 52 international HTA professionals to ICHI and to describe our coder experiences with the ICHI classification. We generally found that ICHI provides a good coverage of PH interventions, despite the challenges we faced due to the lack of detailed reporting in the original survey (5). The broader character of system wide interventions, often involving a variety of institutions and stakeholders, may have presented a hurdle to the application of ICHI coding (including actions, targets and means). Highlighted by the distinction between actions such as advising, education, training, counseling and advocacy, all representing ways to disseminate information to a given audience, and not all survey answers provided a good fit with ICHI coding options. Though the developers of the classification described the complexity level of ICHI as low, the coding of vague distinctions between PHIs presents some challenges (12). Difficulties in the use of ICHI in the field of PHI were also recently reported by Fortune et al. (15). Their study described issues with using ICHI to distinguish between action (“deed done by an actor to the target”) and means (“processes and methods by which the action is carried out”) for interventions delivered in a public health context. In addition, the authors reported that PHI codes often were left unspecified with the appendix ZZ. In addition, there is still a lack of studies using ICHI in the public health sector and its specifics with technology assessments. Based on our experience, we recommend to tailor future surveys more specifically to ensure sufficient data to fit the needs of the ICHI classification. This can be achieved by asking more precise details on the desired effect, actions, targets and means related to PHI. We allowed free text answers to report PHI in our survey and found this contrary to the level of detail needed for ICHI coding.

Our consensual categorization in a double set of independent coding showed that ICHI provides a good coverage of PHIs. We found no interventions that could not be matched with an ICHI code. The good coverage of ICHI with a broad range of interventions was also described by Fortune et al. (10). The study embedded nursing interventions into the ICHI classification, highlighting that only 11 source terms (11%) were found where an appropriate code was missing. To test the coverage of ICHI, the group used 100 high-frequency used nursing interventions. The good coverage of ICHI might be even better due to its updates routinely processed and currently in beta 3 status. In their study, Fortune et al. (10) also stated that the level of familiarity affects the coder agreement (10). Besides training and knowledge about ICHI, we also found the coders' professional background and sex of influence on coding outcomes, although we applied a rigorously independent coding procedure to reduce the influence of individual predispositions. This might be relevant for future studies when setting up a research team, considering a mix of professional backgrounds and sex in a team. However, confirmatory studies would be needed to make this assertion reliable and to evaluate if the difference is truly sex-based or just associated with the professional specialization of the coder. Currently, there are no studies in the literature that evaluated sex-differences in the coding procedures. If similar findings were revealed in the assignment of codes to other types of interventions, it would be important to consider the possible implications. Furthermore, the infrastructure to support ICHIs implementation is yet to be developed. This includes the provision of education and training materials for ICHI and the ability to collect feedback of the user experience (10).

International Classification of Health Interventions sets a standard for conceptualizing and classifying health (care) interventions, it should initiate a worldwide approach to data reporting and pooling of health oriented actions. Standards of reporting on actions, will help to perform benchmarks throughout the different healthcare systems and their potential inequalities. Furthermore, this will likely strengthen insights about the efficiency of primary prevention interventions and thus benefit long-term health of sub-healthy and/or at-risk populations. This is especially relevant to low and lower middle-income countries which spend much larger shares on prevention than upper middle and high income countries (16). Journals might support authors to use ICHI codes in their research reporting, which would streamline peer review, quality measures and rapid processing of high-quality reports.

The ICHI classification provides a common language for health (care) interventions. Health professionals and their viewpoint on health are usually highly rationalized by their medical background and education. International Classification of Health Interventions might create new opportunities to support an inter-sectoral professional viewpoint on PHIs and their often-neglected variance. For instance, hospitals could recalibrate their focus on services toward the integration of more interventions related to the prevention of diseases. Education and advising might become a stronger part of both primary and hospital care, rather than technical -sometimes costly- procedures, such as surgery and pharmaceutical prescriptions. Relatively inexpensive and cost-effective public interventions preserve a neglected potential to tackle the cost burden of aging societies worldwide (17).

From the HTA perspective, ICHI offers several opportunities. The lack of sufficiently solid data in the area of PHI often hampers decisions to adopt or implement a PH technology. Currently, PH interventions represent only a limited proportion of evaluations carried out by institutions engaged in HTA. As mentioned above, the widespread use of ICHI by different stakeholder parties will contribute to a stronger evidence base and thereby enable a higher number of PH technology assessments. In addition, the possibility of categorizing PH-HTA outcomes within a WHO-FIC reference classification will improve global insight in the health-disease continuum and may represent an important aid in health priority setting and policymaking.

The COVID-19 pandemic highlights the importance of those worldwide reporting standards and hopefully, their acceleration. International Classification of Health Interventions provides a promising approach for a common language. If records, research and statistics could be based on ICHI, this would more efficiently contribute to a coherent national and international understanding of health and indications of where actions are required. However, by design, the ICHI codes are not self-explanatory, and the codes do not follow an intuitive hierarchical order. Furthermore, studies have shown that users of WHO classifications are mostly located in North America and Europe (18). Health systems without particular use of comprehensive classification systems will need the support to implement ICHI and reduce the risk of miscoding. Thus, we would like to advise a certain training period for health professionals before coding with ICHI. Concertation with HTA professionals, who play an important role in providing information to health policy makers, may be of added value.

## Conclusions

The ICHI classification provides a language to assist planning and communication about heath (care) interventions across government bodies and health care sectors. A common terminology among HTA professionals and other stakeholders in the health sector is vital to support knowledge sharing across jurisdictions and the evaluation and implementation of effective public health interventions. By providing an organized data structure, ICHI is another component to more harmonized information systems across different areas of policy and services. International Classification of Health Interventions has the potential to contribute to the appraisal of public health interventions across national and international settings and the initiatives to collect real world data about their effectiveness. However, a certain training period on the handling of such a comprehensive terminology is essential to release the full potential of ICHI. Our research shows that the application of ICHI on public health data is demanding, even for researchers with experience in data management and with an advanced educational background. Based on our experience, we would recommend to tailor future surveys more specifically to the needs of the classification and we advise training for health professionals before coding with ICHI.

## Data Availability Statement

The raw data supporting the conclusions of this article will be made available by the authors, without undue reservation.

## Author Contributions

MW, SG, JS, LE, and IL-W performed the coding of PH interventions. MW wrote the sections of the manuscript including the first draft. IG-I, IL-W, and MW contributed to the conception and design of the study. JS, LE, and SG extracted the survey data and performed the statistical analysis. All the authors contributed to manuscript revision as well as read and approved the submitted version.

## Conflict of Interest

The authors declare that the research was conducted in the absence of any commercial or financial relationships that could be construed as a potential conflict of interest.
