# Supplementary material for: Coding Public Health Interventions for Health Technology Assessments: A Pilot Experience With WHO's International Classification of Health Interventions (ICHI)
Source: Front Public Health. 2021 Jun 16;9:620637. doi: 10.3389/fpubh.2021.620637 (PMC8242241; doi:10.3389/fpubh.2021.620637)
Supplement: Supplementary file 1 [file Table_1.DOCX]

SUPPLEMENTARY TABLE

| # | PH Intervention candidate | ICHI coding options | ICHI Descriptor |
| --- | --- | --- | --- |
| 1 | *Down syndrome screening* | NMR.AA.BJ | Ultrasound assesment for detection of fetal abnormality |
|  |  | NMR.AA.ZZ | Assessment of fetal or embryonic structure |
| 2 | *Pre-/postnatal mother-child care* | SSK.PM.ZZ | Education about parent-child relationships |
|  |  | SSK.RB.ZZ | Practical support for parent-child relationships |
|  |  | VEJ.PH.ZZ | Training to influence parenting behaviours |
|  |  | VEJ.PM.ZZ | Education to influence parenting behaviours |
|  |  | VEJ.PN.ZZ | Advising about parenting behaviours |
|  |  | VEJ.PP.ZZ | Counselling about parenting behaviours |
|  |  | VEJ.RC.ZZ | Emotional support for parenting behaviours |
|  |  | VEJ.VB.ZZ | Awareness raising to influence parenting behaviours |
| 3 | *Screening for tyrosinaemia in newborns* | ET2.AA.ZZ | Assessment of functions of the metabolic and endocrine systems |
|  |  | ET2.AC.ZZ | Test of functions of the metabolic and endocrine systems |
|  |  | PND.AH.XA | Blood collection from heel |
| 4 | *HPV screening* | NMF.AH.AC | Cervical papanicolaou smear |
|  |  | UBC.VC.ZZ | Population public health surveillance |
|  |  | VDB.WG.QF | Economic incentives to encourage improved health behaviours relating to use of health screening services |
|  |  | VDB.VC.ZZ | Public health surveillance concerning screening behaviours |
|  |  | VEF.VC.ZZ | Public health surveillance concerning sexual behaviours |
| 5 | *Parent-Child Assistance Program for Preventing Fetal Alcohol Spectrum Disorder* | VAA.PH.ZZ | Training to influence alcohol use behaviours |
|  |  | VAA.PM.ZZ | Education to influence alcohol use behaviours |
|  |  | VAA.PN.ZZ | Advising about alcohol use behaviours |
|  |  | VAA.PP.ZZ | Counselling about alcohol use behaviours |
| 6 | *Prep Exposition* | UE1.VP.ZZ | Improving access to services |
|  |  | VEF.AA.ZZ | Assessment of sexual behaviours |
|  |  | VEF.RD.ZZ | Provision of products to support improved sexual behaviours |
|  |  | VEF.VA.ZZ | Capacity buidling interventions targeting sexual behaviours |
|  |  | VEF.VC.ZZ | Public health surveillance concerning sexual behaviours |
| 7 | *Hemophilus influenzae type b vaccine* | DTB.DB.AE | Percutaneous administration of immunological agent |
|  |  | UAC.AA.ZZ | Assessment of medication |
| 8 | *pre-exposure prophylaxis (PrEP)* | DTB.AM.ZZ | Observation of functions of the immunological system |
|  |  | DTB.DB.AC | Oral immunisation |
|  |  | UAC.AA.ZZ | Assessment of medication |
|  |  | UAC.PM.ZZ | Education about medication |
|  |  | UAC.PN.ZZ | Advising about medication |
|  |  | UAC.RB.ZZ | Practical support with medication |
| 9 | *Community-based Primary Care (evaluated three times in total in 2015, 2016, 2017)* | UA1.VC.ZZ | Public health surveillance concerning products and technology |
|  |  | UE1.VC.ZZ | Public health surveillance concerning services, systems and policies |
|  |  | UE1.VP.ZZ | Improving access to services |
|  |  | UEP.AA.ZZ | Assessment of health services, systems and policies |
| 10 | *Autologous chondrocyte implantation* | MMC.KD.AB | Arthroscopic transplantation of chondrocyte cells of knee joint |
|  |  | MMC.KD.AE | Pecutaneous transplantation of chondrocyte cells of knee joint |
| 11 | *sport* | VEB.RD.ZZ | Provision of products to support improved physical activity behaviours |
|  |  | VEB.RF.ZZ | Providing opportunities for participation in relation to physical activity behaviours |
|  |  | VEB.TI.ZZ | Prescription for physical activity behaviours |
| 12 | *Imaging modalities in breast cancer screening* | LCA.BA.BA | Mammography |
|  |  | LCA.BA.BB | Contrast radiogram of mammary ducts |
|  |  | UA1.VC.ZZ | Public health surveillance concerning products and technology |
| 13 | *Intervention promoting social and emotionnal skills among children aged 2-6* | VEK.PH.ZZ | Training to influence social behaviours |
|  |  | VEK.PM.ZZ | Education to influence social behaviours |
|  |  | VEK.VB.ZZ | Awareness raising to influence social behaviours |
| 14 | *Pulmonar Tuberculosis in homeless people* | JBF.BA.BA | X-ray of lung, not elsewhere classified |
|  |  | UA1.WG.QF | Economic incentives concerning products and technology in relation to health |
|  |  | UBC.VC.ZZ | Public health surveillance concerning population |
|  |  | UE1.VC.ZZ | Public health surveillance concerning services, systems and policies |
|  |  | UEP.VE.ZZ | Health care infection control measures |
| 15 | *High Risk Breast Cancer Surveillance* | LCA.AE.AH | Manual examination of breast |
|  |  | LCA.BA.BA | Mammography |
|  |  | LCA.BA.BH | Magnetic resonance imaging of breast |
|  |  | LCA.BA.BJ | Breast ultrasonography |
|  |  | VDB.WG.QF | Economic incentives to encourage improved health behaviours relating to use of health screening services |
|  |  | UBC.VC.ZZ | Population public health surveillance |
|  |  | PYA.PP.ZZ | Genetic counselling |
| 16 | *Human papillomavirus vaccination* | DTB.DB.AE | Percutaneous administration of immunological agent |
|  |  | UBC.VC.ZZ | Population public health surveillance |
|  |  | VDA.VC.ZZ | Public health surveillance concerning immunisation behaviours |
| 17 | *Flu vaccination* | DTB.DB.AE | Percutaneous administration of immunological agent |
|  |  | JT2.PP.ZZ | Counselling for functions of the respiratory system |
|  |  | UBC.VC.ZZ | Population public health surveillance |
|  |  | VDA.VC.ZZ | Public health surveillance concerning immunisation behaviours |
| 18 | *Screening chlamydia trachomatis infection* | VEF.AA.ZZ | Assessment of sexual behaviours |
|  |  | VEF.PN.ZZ | Advising about sexual behaviours |
|  |  | VDB.VC.ZZ | Public health surveillance concerning screening behaviours |
|  |  | VEF.VC.ZZ | Public health surveillance concerning sexual behaviours |
| 19 | *Recommendation on best practices of prevention and intervention in the fugues by young people in rehabilitation center for young people in difficulty of adaptation* | UEO.PM.ZZ | Education about general social support services, systems and policies |
|  |  | UEP.PM.ZZ | Education about health services, systems and policies |
|  |  | UEP.PN.ZZ | Advising about health services, systems and policies |
| 20 | *Tobacco prevention (electronic cigarettes and snus)* | VAB.AA.ZZ | Assessment of tobacco use behaviours |
|  |  | VAB.PN.ZZ | Advising about tobacco use behaviours |
|  |  | VAB.RD.ZZ | Provision of products to support improved health behaviours relating to tobacco use |
| 21 | *Fibroscan* | KCA.BA.BE | Liver scan and radioisotope function study |
|  |  | KCA.BA.BJ | Ultrasound of liver |
| 22 | *HTA for pneumococcal vaccines for children below five years old* | DTB.DB.AE | Percutaneous administration of immunological agent |
|  |  | VDA.WG.QF | Economic incentives to encourage improved health behaviours relating to use of immunisation services |
|  |  | UEP.VE.ZZ | Health care infection control measures |
|  |  | VDA.VC.ZZ | Public health surveillance concerning immunisation behaviours |
| 23 | *Individual Health Status Assessment using e-health and community workers* | UA1.VC.ZZ | Public health surveillance concerning products and technology |
|  |  | UBC.VC.ZZ | Public health surveillance concerning population |
|  |  | VAF.VC.ZZ | Public health surveillance concerning digital technology use behaviours |
|  |  | VFX.VC.ZZ | Public health surveillance concerning other health-related behaviours |
| 24 | *Colorectal cancer screening for population at risk* | KBP.AE.AD | Colonoscopy |
|  |  | KBP.BA.BC | Computerised tomography of colon, not elsewhere classified |
|  |  | KBU.AE.AD | Sigmoidoscopy |
|  |  | UA1.VC.ZZ | Public health surveillance concerning products and technology |
|  |  | VDB.VC.ZZ | Public health surveillance concerning screening behaviours |
| 25 | *HPV-vaccination* | DTB.DB.AE | Percutaneous administration of immunological agent |
|  |  | UA1.VC.ZZ | Public health surveillance concerning products and technology |
|  |  | VEF.VC.ZZ | Public health surveillance concerning sexual behaviours |
| 26 | *Screening for cystic fibrosis in newborns* | JT2.AA.ZZ | Assessment of functions of the respiratory system |
|  |  | PND.AH.XA | Blood collection from heel |
|  |  | PYA.PP.ZZ | Genetic counselling |
| 27 | *STI screening during pregnancy* | NUE.ZY.ZZ | Other interventions on functions related to pregnancy, not elsewhere classified |
|  |  | VEF.AA.ZZ | Assessment of sexual behaviours |
|  |  | VEF.PP.ZZ | Counselling about sexual behaviours |
|  |  | VEF.VC.ZZ | Public health surveillance concerning sexual behaviours |
| 28 | *Housing improvements* | UAM.TD.ZZ | Collaborating or building partnerships in relation to the built environment, including housing |
|  |  | UAM.TK.ZZ | Public facilities or infrastructure development to improve aspects of the built environment, including housing |
|  |  | UAM.VA.ZZ | Capacity building interventions targeting the built environment, including housing |
|  |  | UAM.VC.ZZ | Public health surveillance concerning the built environment, including housing |
| 29 | *Rotavirus Vaccine* | DTB.DB.AE | Percutaneous administration of immunological agent |
|  |  | VDA.VC.ZZ | Public health surveillance concerning immunisation behaviours |
| 30 | *Lung cancer screening* | JBF.BA.BB | Radiography of lung with contrast |
|  |  | JBF.BA.BH | Magnetic resonance imaging of lung |
|  |  | PAE.BA.BC | Computerised tomography of thorax, not elsewhere classified |
|  |  | PAE.BA.BD | Computerised tomography of thorax with intravenous contrast |
|  |  | UBC.VC.ZZ | Population public health surveillance |
|  |  | VDB.VC.ZZ | Public health surveillance concerning screening behaviours |
| 31 | *Interventions preventing cannabis use among high school* | VA1.AA.ZZ | Assessment of substance-related and addictive behaviours |
|  |  | VA1.AC.ZZ | Test of substance -related and addictive behaviours |
|  |  | VA1.PN.ZZ | Advising about substance-related and addictive behaviours |
|  |  | VA1.VC.ZZ | Public health surveillance concerning substance-related and additive behaviours |
|  |  | VAC.AC.ZZ | Test of illicit drug use behaviours |
|  |  | VAC.PH.ZZ | Training to influence illicit drug use behaviours |
|  |  | VAC.PM.ZZ | Education to influence illicit drug use behaviours |
|  |  | VAC.PN.ZZ | Advising about illicit drug use behaviours |
|  |  | VAC.PP.ZZ | Counselling about illicit drug use behaviours |
|  |  | VAC.TD.ZZ | Collaborating or building partnerships in relation to illicit drug use behaviours |
|  |  | VAC.TM.ZZ | Environment modification to influence illicit drug use behaviours |
|  |  | VAC.VA.ZZ | Capacity building interventions targeting illicit drug use behaviours |
| 32 | *Programs to prevent obesity in adolescents* | KTN.PN.ZZ | Advising about functions related to weight management |
|  |  | KTN.PP.ZZ | Counselling for weight management |
|  |  | KTN.RB.ZZ | Practical support for weight management |
|  |  | KTN.RC.ZZ | Emotional support for weight management |
|  |  | VEA.PH.ZZ | Training to influence eating behaviours |
|  |  | VEA.PM.ZZ | Education to influence eating behaviours |
|  |  | VEA.PN.ZZ | Advising about eating behaviours |
|  |  | VEA.RD.ZZ | Provision of products to support improved eating behaviours |
|  |  | VEA.VB.ZZ | Awareness raising to influence eating behaviours |
|  |  | VEA.VC.ZZ | Public health surveillance concerning eating behaviours |
| 33 | *Public Access Defibrillation* | HTB.SC.AH | Conversion of cardiac rhythm |
|  |  | UA1.VB.ZZ | Awareness raising concerning products and technology |
|  |  | UA1.VC.ZZ | Public health surveillance concerning products and technology |
|  |  | UA1.WG.QF | Economic incentives concerning products and technology in relation to health |
| 34 | *Smallpox vaccination* | DTB.DB.AE | Percutaneous administration of immunological agent |
|  |  | UEP.VE.ZZ | Health care infection control measures |
|  |  | VFX.PP.ZZ | Counselling about other health-related behaviours |
|  |  | VFX.VB.ZZ | Awareness raising to influence other health-related behaviours |
|  |  | VDA.VC.ZZ | Public health surveillance concerning immunisation behaviours |
| 35 | *Vaccination program* | DTB.DB.AE | Percutaneous administration of immunological agent |
|  |  | VDA.VC.ZZ | Publich health surveillance concerning immunisation behaviours |
| 36 | *Price regulations on health affecting consumer goods* | UAA.WG.QF | Economic incentives concerning products or substances for personal consumption in relation to health |
|  |  | UAA.WJ.ZZ | Policy change concerning products or substances for personal consumption |
|  |  | VEA.WG.QF | Economic incentives to encourage improved health behaviours relating to eating |
|  |  | VEA.WJ.ZZ | Policy change concerning eating behaviours |
| 37 | *Eficacia, seguridad y evaluación económica de la mamografía digital directa (dr-m) contra la mamografía digital indirecta o computada (cr-m)* | LCA.BA.BA | Mammography |
|  |  | LCA.BA.BH | Magnetic resonance imaging of breast |
|  |  | LCA.BA.BJ | Breast ultrasonography |
|  |  | UA1.AA.ZZ | Assessment of products and technology |
|  |  | UA1.VC.ZZ | Public health surveillance concerning products and technology |
|  |  | UA1.WG.QF | Economic incentives concerning products and technology in relation to health |
| 38 | *Cities changing diabetes* | VE1.TM.ZZ | Environment modification to influence lifestyle behaviours |
|  |  | VE1.VA.ZZ | Capacity building interventions targeting lifestyle behaviours |
|  |  | VE1.TK.ZZ | Public facilities or infrastructure development to support improved lifestyle behaviours |
| 39 | *Cervical cancer screening* | NMF.AD.AC | Cervical biopsy |
|  |  | NMF.AH.AC | Cervical papanicolaou smear |
|  |  | UBC.VC.ZZ | Population public health surveillance |
|  |  | VDB.VC.ZZ | Public health surveillance concerning screening behaviours |
|  |  | VDB.PN.ZZ | Advising about screening behaviours |
|  |  | VEF.VC.ZZ | Public health surveillance concerning sexual behaviours |
| 40 | *adolescent screening* | SDJ.AA.ZZ | Assessment of handling stress and other psychological demands |
|  |  | SDJ.AA.ZZ | Assessment of handling stress and other psychological demands |
|  |  | SDJ.AC.ZZ | Test of handling stress and other psychological demands |
|  |  | SDJ.AN.ZZ | Interview in relation to handling stress and other psychological demands |
| 41 | *screening for depression* | SDJ.AA.ZZ | Assessment of handling stress and other psychological demands |
|  |  | SDJ.AC.ZZ | Test of handling stress and other psychological demands |
|  |  | SDJ.AN.ZZ | Interview in relation to handling stress and other psychological demands |
|  |  | VDB.VC.ZZ | Public health surveillance concerning screening behaviours |
| 42 | *Hep C screening* | DTB.AA.ZZ | Assessment of immunological system functions |
|  |  | VDB.VC.ZZ | Public health surveillance concerning screening behaviours |
|  |  | VDB.WG.QF | Economic incentives to encourage improved health behaviours relating to use of health screening services |
| 43 | *Thrombophilia testing-inherited to prevent VTE* | IZZ.AA.ZZ | Assessment of blood vessel, not elsewhere classified |
|  |  | PYA.PP.ZZ | Genetic counselling |
| 44 | *Air pollution text alerts* | UB1.VB.ZZ | Awareness raising concerning aspects of the natural environment and human-made changes to environment |
|  |  | UB1.VF.ZZ | Health alert concerning aspects of the natural environment and human-made changes to the environment |
|  |  | UBM.RD.ZZ | Provision of products or technology to reduce or protect against outdoor air pollution |
|  |  | UBM.VB.ZZ | Awareness raising concerning outdoor air quality |
| 45 | *Human Papilloma Virus Vaccine* | DTB.DB.AE | Percutaneous administration of immunological agent |
|  |  | VDA.VC.ZZ | Public health surveillance concerning immunisation behaviours |
| 46 | *Pertinence of prostate cancer screening* | NGA.BA.BA | X-ray of prostate and seminal vesicles |
|  |  | NGA.BA.BJ | Ultrasound of prostate |
|  |  | VDB.VC.ZZ | Public health surveillance concerning screening behaviours |
|  |  | KBX.AE.AC | Digital examination of anus |
| 47 | *Atrial Fibrillation Screening* | HTB.AA.ZZ | Assessment of heart functions |
|  |  | HTB.AI.AH | Cardiac monitoring |
|  |  | VDB.VC.ZZ | Public health surveillance concerning screening behaviours |
|  |  | VDB.WG.QF | Economic incentives to encourage improved health behaviours relating to use of health screening services |
| 48 | *CT calcium scoring* | HIA.BA.AF | Intravascular imaging of coronary vessels |
|  |  | HZZ.BA.BD | Computerised tomography of heart with intravenous contrast medium |
|  |  | VDB.VC.ZZ | Public health surveillance concerning screening behaviours |
| 49 | *Tomosynthese (mammography) in breast cancer screening* | LCA.BA.BA | Mammography |
|  |  | UA1.AA.ZZ | Assessment of products and technology |
|  |  | UA1.VC.ZZ | Public health surveillance concerning products and technology |
| 50 | *Screening for abdominal aortic aneurysm* | HIF.BA.BJ | Aortic arch ultrasonography |
|  |  | HZZ.BA.BJ | Ultrasound of heart |
|  |  | VDB.WG.QF | Economic incentives to encourage improved health behaviours relating to use of health screening services |
|  |  | VDB.VC.ZZ | Public health surveillance concerning screening behaviours |
| 51 | *HTA for smoking cessation pharmaceutical products* | UA1.WG.QF | Economic incentives concerning products and technology in relation to health |
|  |  | VAB.RD.ZZ | Provision of products to support improved health behaviours relating to tobacco use |
|  |  | VAB.VC.ZZ | Public health surveillance concerning tobacco use behaviours |
| 52 | *Technologies for tobacco cesation* | VAB.PM.ZZ | Education to influence tobacco use behaviours |
|  |  | VAB.PN.ZZ | Advising about tobacco use behaviours |
|  |  | VAB.RD.ZZ | Provision of products to support improved health behaviours relating to tobacco use |
|  |  | VAB.VC.ZZ | Public health surveillance concerning tobacco use behaviours |
| 53 | *Screening for hepatitis B and C* | DTB.AA.ZZ | Assessment of immunological system functions |
|  |  | VDB.VC.ZZ | Public health surveillance concerning screening behaviours |
| 54 | *Water fluoridation* | UBN.AA.ZZ | Assessment of water quality |
|  |  | UBN.VC.ZZ | Public health surveillance concerning water quality |
|  |  | UBN.VI.ZZ | Water fortification |
|  |  | UBN.VI.ZZ | Water fortification |
| 55 | *Take home naloxone distributed through emergency departments and ambulances* | UA1.VC.ZZ | Public health surveillance concerning products and technology |
|  |  | UAC.AA.ZZ | Assessment of medication |
|  |  | UAC.AA.ZZ | Assessment of medication |
| 56 | *Intensified Polio Eradication Program* | DTB.DB.AE | Percutaneous administration of immunological agent |
|  |  | UEP.VE.ZZ | Health care infection control measures |
| 57 | *HPV testing for cervix cancer screening* | NMF.AH.AC | Cervical papanicolaou smear |
|  |  | VDB.VC.ZZ | Public health surveillance concerning screening behaviours |
| 58 | *BCG Vaccination for children at high-risk of acquiring TB* | DTB.DB.AE | Percutaneous administration of immunological agent |
|  |  | UA1.WG.QF | Economic incentives concerning products and technology in relation to health |
| 59 | *Blood and stool biomarker testing* | VDB.VC.ZZ | Public health surveillance concerning screening behaviours |
|  |  | DIA.JH.AF | Collection of blood or blood products |
|  |  | PZX.AH.XG | Specimen collection, faeces |
|  |  | PYA.PP.ZZ | Genetic counselling |
| 60 | *Search for human papillomavirus (HPV) in uterus cancer screening* | NMF.AH.AC | Cervical papanicolaou smear |
|  |  | VDB.VC.ZZ | Public health surveillance concerning screening behaviours |
| 61 | *Prevention of substance abuse among children and adolescents* | VA1.VC.ZZ | Public health surveillance concerning substance-related and additive behaviours |
|  |  | VAC.PM.ZZ | Education to influence illicit drug use behaviours |
|  |  | UAA.WG.QF | Economic incentives concerning products or substances for personal consumption in relation to health |
| 62 | *Screening for cancer (e.g. mammography; PSA)* | VDB.VC.ZZ | Public health surveillance concerning screening behaviours |
| 63 | *Chronic Disease Self-Management* | SMH.AA.ZZ | Assessment of looking after one's health |
|  |  | SMH.AA.ZZ | Assessment of looking after one's health |
|  |  | UA1.WG.QF | Economic incentives concerning products and technology in relation to health |
|  |  | UBC.VC.ZZ | Public health surveillance concerning population |
|  |  | UE1.VC.ZZ | Public health surveillance concerning services, systems and policies |
| 64 | *Screening instruments for risk of suicide* | VBA.AA.ZZ | Assessment of self-harm behaviours |
|  |  | VBA.VC.ZZ | Public health surveillance concerning self-harm behaviours |
| 65 | *Screening for cervical carcinoma* | NMF.AH.AC | Cervical papanicolaou smear |
|  |  | VDB.VC.ZZ | Public health surveillance concerning screening behaviours |
| 66 | *National AIDS Control Program Interventions* | DTB.AA.ZZ | Assessment of immunological system functions |
|  |  | VEF.PH.ZZ | Training to influence sexual behaviours |
|  |  | VEF.PM.ZZ | Education to influence sexual behaviours |
|  |  | VEF.RD.ZZ | Provision of products to support improved sexual behaviours |
|  |  | VEF.VB.ZZ | Awareness raising to influence sexual behaviours |
|  |  | VEF.VC.ZZ | Public health surveillance concerning sexual behaviours |
| 67 | *Smoking cessation interventions* | VAB.RD.ZZ | Provision of products to support improved health behaviours relating to tobacco use |
|  |  | VAB.VC.ZZ | Public health surveillance concerning tobacco use behaviours |
|  |  | VAB.WG.QF | Economic incentives to encourage improved health behaviours relating to use of health screening services |
| 68 | *Screening for colorectal carcinoma* | KBP.AE.AD | Colonoscopy |
|  |  | VDB.VC.ZZ | Public health surveillance concerning screening behaviours |
|  |  | PZX.AH.XG | Specimen collection, faeces |
| 69 | *Mobile health application for community health workers* | UA1.AA.ZZ | Assessment of products and technology |
|  |  | VAF.VC.ZZ | Public health surveillance concerning digital technology use behaviours |
|  |  | VAF.VA.ZZ | Capacity building interventions targeting digital technology use behaviours |
| 70 | *HPV testing for cervical cancer screening* | NMF.AH.AC | Cervical papanicolaou smear |
|  |  | VDB.VC.ZZ | Public health surveillance concerning screening behaviours |
|  |  | VDB.WG.QF | Economic incentives to encourage improved health behaviours relating to use of health screening services |
| 71 | *screening for thalassaemia* | DTA.AA.ZZ | Assessment of haematological system functions |
|  |  | PYA.PP.ZZ | Genetic counselling |
|  |  | VDB.VC.ZZ | Public health surveillance concerning screening behaviours |
| 72 | *Schools-based programme of HPV vaccination for boys* | DTB.DB.AE | Percutaneous administration of immunological agent |
|  |  | VDA.WG.QF | Economic incentives to encourage improved health behaviours relating to use of immunisation services |
|  |  | VDA.VC.ZZ | Public health surveillance concerning immunisation behaviours |
| 73 | *Nurse Mentoring Program* | SBQ.PH.ZZ | Training in acquiring skills |
|  |  | SA1.RB.ZZ | Practical support with learning and applying knowledge |
|  |  | SC2.PH.ZZ | Training in applying knowledge |
| 74 | *Pre-Exposure Prophylaxis (PrEP) programme to prevent HIV in Ireland* | DTB.DB.AC | Oral immunisation |
|  |  | UAC.PM.ZZ | Education about medication |
|  |  | UAC.PN.ZZ | Advising about medication |
|  |  | UAC.RB.ZZ | Practical support with medication |
|  |  | VDA.VC.ZZ | Public health surveillance concerning immunisation behaviours |
|  |  | VEF.AA.ZZ | Assessment of sexual behaviours |
| 75 | *Screening of cervical cancer, diabetes and hypertension in India* | VDB.VC.ZZ | Public health surveillance concerning screening behaviours |
